# Host and pathogen factors modulate tuberculosis clinical severity independent of molecular bacterial burden: insights from a multicenter Ghanaian cohort

**DOI:** 10.1016/j.ijregi.2026.100937

**Published:** 2026-06-27

**Authors:** Theophilus Afum, Prince Asare, Stephen Osei-Wusu, Ivy Naa Koshie Lamptey, Susan Darkwahene-Boateng, Britta S. Meyer, Tobias L. Lenz, Dorothy Yeboah-Manu

**Affiliations:** 1 Noguchi Memorial Institute for Medical Research, College of Health Sciences, University of Ghana, Legon, Ghana; 2 West African Centre for Cell Biology of Infectious Pathogens, University of Ghana, Legon, Ghana; 3 Research Unit for Evolutionary Immunogenomics, Department of Biology, University of Hamburg, Hamburg, Germany

**Keywords:** Tuberculosis, Bacterial load, Host–pathogen interaction, GeneXpert, Bandim TB score, Disease severity

## Abstract

**Objectives:**

Tuberculosis (TB) exhibits marked clinical heterogeneity, complicating prognosis, and disease management. Identifying reliable predictors of disease trajectory and severity could improve risk stratification.

**Methodology:**

We investigated determinants of TB severity in 727 sputum-positive individuals recruited from five health facilities in Ghana. Participants were classified as TB-only, TB–human immunodeficiency virus, or TB–diabetes mellitus. Disease severity was assessed using the Bandim TB score II. Bacterial burden was quantified using GeneXpert *Mycobacterium tuberculosis*/rifampicin Ultra cycle threshold values, sputum smear microscopy grades, and time to culture positivity. Multivariable regression models were used to evaluate host, pathogen, and clinical determinants of severity.

**Results:**

Bacterial load differed modestly across comorbidity groups, but GeneXpert cycle threshold values showed no significant correlation with clinical severity (Spearman’s ρ = –0.006, *P* = 0.894). Lower body mass index was consistently associated with increased odds of severe TB (odds ratio = 0.639, 95% confidence interval 0.566-0.714; *P* = 3.7 × 10^−14^). Self-reported Ewe ethnicity was associated with milder clinical severity. Higher sputum smear grade was independently associated with more severe disease.

**Conclusion:**

Host nutritional status, socio-demographic factors, and pathogen genotype were more consistently associated with TB severity than molecular bacterial burden alone, supporting a host–pathogen interaction framework to explain clinical heterogeneity and improve management.

## Highlights

• Host nutritional status (body mass index) independently predicts tuberculosis (TB) severity, irrespective of molecular bacterial load.
• Socio-demographic factors, including self-reported ethnicity, are associated with differential TB severity.
• TB–human immunodeficiency virus and TB–diabetes comorbidities modify clinical severity patterns at presentation.
• Host–pathogen interactions better explain TB clinical heterogeneity than molecular bacterial burden alone.

## Introduction

Tuberculosis (TB) caused by *Mycobacterium tuberculosis* (MTB) complex (MTBC) remains the leading cause of death by a single infectious disease globally [1]. Despite advancements in standardized treatment regimens, patient outcomes vary widely, reflecting heterogeneity in host immune responses, comorbidities, pathogen diversity, and socio-demographic factors [2].

The MTBC comprises multiple lineages with distinct geographical distributions and host adaptations, which have been associated with differences in transmission and clinical presentation [3]. Lineage diversity, along with host immune and genetic variability, contributes to clinical heterogeneity; however, the extent to which the bacterial burden explains severity remains uncertain. Moreover, the prevalence of comorbidities, such as human immunodeficiency virus (HIV) and type 2 diabetes (DM), is known to modulate the progression of TB and contribute to interindividual variability in TB severity, both of which exacerbate TB disease [4].

Molecular diagnostic platforms, such as Cepheid’s GeneXpert MTB/rifampicin (RIF) Ultra assay, rapidly quantify the MTBC bacterial load. This multiplex polymerase chain reaction (PCR)-based assay identifies MTBC infection, while targeting the *rpoB* gene can determine rifampicin resistance. The test uses cycle threshold (Ct) values, which indicate the number of PCR cycles required for a fluorescent signal from amplified MTBC DNA to exceed a predefined background threshold, reflecting bacterial load and, in some contexts, TB severity [5]. However, bacterial load alone may not accurately capture the heterogeneity in TB disease. To complement this shortcoming, scoring systems such as the Bandim TB score and Karnofsky Performance score [[6], [7], [8]], which integrate symptom-based and anthropometric parameters, could provide a broader measure of disease burden, particularly in resource-limited settings.

Despite these advances, it remains unclear how host comorbidities, self-reported ethnicity, and pathogen lineage collectively shape bacterial burden and TB clinical severity. Ghana provides a unique setting for studying TB heterogeneity due to the coexistence of both modern and ancient lineages with a growing number of comorbidities such as HIV and diabetes. By integrating bacterial load metrics (GeneXpert Ct values, sputum microscopy, and time to culture positivity) with host characteristics (body mass index [BMI], comorbidity, and self-reported ethnicity) and pathogen genotypes, we aimed to identify key predictors of disease severity using multivariate regression frameworks.

## Methods

### Study design and recruitment

**Figure 1:**
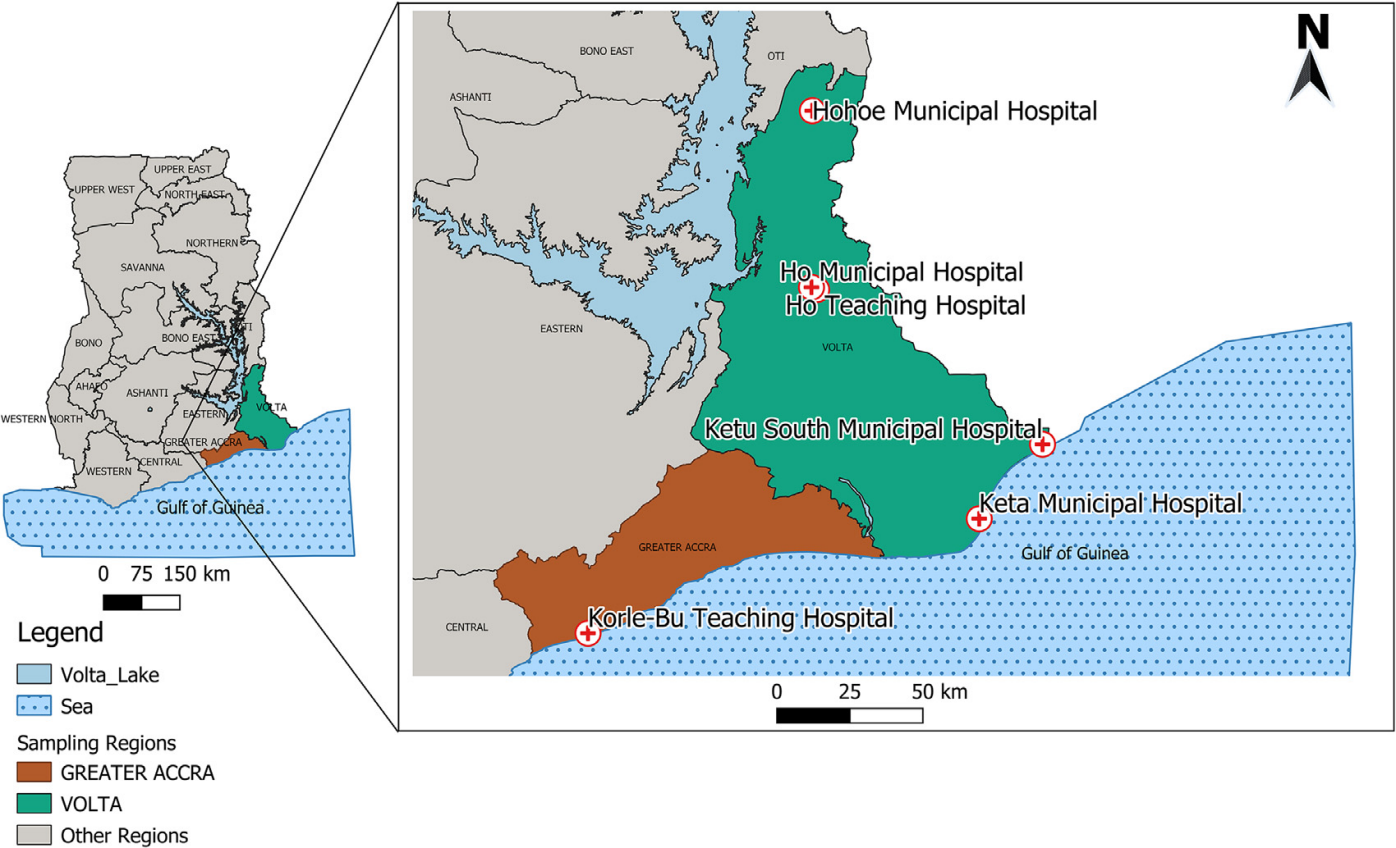
A map showing the different health facilities of the study. A map showing the different health facilities of the study. The figure shows the five sampling sites used in this study. These were health facilities in the Greater Accra and Volta Regions of Ghana. Map was constructed using QGIS Version 3.14.16-Pi (QGIS Development Team, 2020. QGIS Geographic Information System. Open Source Geospatial Foundation Project. http://qgis.org).

We conducted a cross-sectional study of newly diagnosed pulmonary TB cases recruited prospectively from five health facilities in southern Ghana from October 2022 to December 2024 (Figure 1). Given the absence of longitudinal follow-up, associations between host factors and disease severity were interpreted as non-causal.

### Inclusion and exclusion criteria

Only newly GeneXpert-confirmed TB participants who either had not started anti-TB medication or had been on anti-TB medication for less than 2 weeks were included in this study. Patients with prior anti-TB treatment exceeding two weeks were excluded.

### TB cohorts

Three main cohorts were included in the study. Firstly, TB-only: These were participants who were only infected with TB and had no comorbidities. TB-HIV: participants who were confirmed microbiologically as having both TB and HIV. TB-DM: participants with confirmed TB and diabetes mellitus type 2 (these were confirmed diabetic patients usually on diabetic treatment). HIV status was determined using the Oraquick HIV self-test kit (OraSure Technologies, USA), while diabetes status was confirmed using fasting blood glucose levels >7 mmol/L on the Sinocare Gold Accu glucometer and HbA1c ≥6.5%.

### Sample collection

Sputum samples were collected from GeneXpert MTB/RIF Ultra confirmed pulmonary TB cases into a wide-mouth container, appropriately labeled and parafilm-sealed before cold chain transportation to Noguchi Memorial Institute for Medical Research (NMIMR) for processing. Samples were processed within 24 hours of receipt to minimize degradation and maintain sample integrity.

### Molecular bacterial load assessment

Expectorated sputum from suspected TB cases was processed using Cepheid’s GeneXpert MTB/RIF Ultra Assay (Cepheid, Sunnyvale, CA) [9]. This PCR-based assay detects MTBC and resistance to rifampicin by targeting the insertion sequences IS*6110* and IS*1081* and the *rpoB* gene, respectively. Bacterial load was estimated using quantitative Ct values. Data analysis was performed using GX Software version 4.3, which categorizes bacterial load as high (Ct < 16), medium (Ct = 16-22), low (Ct = 22-28), very low (Ct > 28), trace (Ct ≥ 35). Ct values for each of the positive samples were exported for downstream analysis. Trace results were excluded from correlation analysis. GeneXpert MTB/RIF Ultra Ct values were used as a semi-quantitative proxy for bacterial load. Ct values may be influenced by sputum quality and variation in *IS*6110 copy number across MTBC lineages.

### Sputum culture and MTBC characterization

Sputum samples were decontaminated with 5% oxalic acid as previously described [10] and inoculated on four Lowenstein-Jensen slants (LJ); two glycerol and two pyruvate-enriched. The inoculated LJ slants were incubated at 37°C and monitored for growth for up to 12 weeks. Time to positivity (TTP) was defined as the number of days until visible mycobacterial growth.

MTBC isolates were characterized using spoligotyping and whole-genome sequencing. Briefly, genotyping of the isolates to the lineage and sub-lineage level was performed by following the lineage and strain classification of the MTBC in a stepwise manner using the procedures as previously described [11]. The spoligotyping patterns and assigned shared type numbers were obtained as defined according to the SITVITWEB database [12].

### Sputum smear microscopy

Smears prepared from decontaminated sediments were stained using the Ziehl-Neelsen technique and graded using WHO’s recommended grading system: Negative, Scanty, 1+, 2+, and 3+ [13].

### Assessment of TB severity

Clinical severity was assessed using the Bandim TB score II, which integrates symptom-based and clinical parameters, including cough, dyspnea, chest pain, anemia, temperature ≥37.5°C, BMI, and mid-upper-arm circumference. Each symptom was scored as 1 when present and 0 when absent. BMI was scored as 1 (<18 kg/m²), 2 (<16 kg/m²), or 0 (≥18 kg/m²). These individual scores for each symptom are then added to form a total score. These total scores are then recorded as severity classes (SC). Three severity classes, mild (SCI = 0-4), moderate (SCII = 5-6), and severe (SCIII ≥7) were used [6]. Bandim TB score II was chosen for its proven reliability in resource-limited settings and usage in cross-sectional study designs [7].

### Data collection and analysis

All demographic, diagnostic, and clinical data were collected using a standardized case recruitment form. These data were double entered into a Microsoft Access Database. All statistical analyses were conducted in R (version 4.4.0). Continuous variables were summarized as medians and interquartile ranges or means, while categorical variables were summarized as frequencies and percentages. ANOVA or Kruskal-Wallis tests were used for group comparisons. Pairwise comparisons were performed using the Wilcoxon rank-sum test with Bonferroni correction for multiple comparisons.

Multivariable linear, ordinal logistic, and multinomial logistic regression models were fitted to identify independent predictors of TB severity. Models were adjusted for age, sex, comorbidity, BMI, MTBC lineage, and self-reported ethnicity. Multicollinearity was assessed using the generalized variance inflation factor (GVIF); all predictors had GVIF^(1/(2*df))^ < 2.24, indicating no problematic collinearity (equivalent to VIF <5 for continuous predictors). For the ordinal logistic regression model, the proportional-odds assumption was evaluated using the Brant test. The omnibus test showed no overall violation (χ² = 16.09, df = 11, *P* = 0.138). BMI showed a predictor-level violation (χ² = 8.80, df = 1, *P* = 0.003), consistent with its structural role as a direct scoring component of TB score II; BMI results from the ordinal model are therefore interpreted alongside the sensitivity analyses described below. To address the sparse severe category (n = 17 in the analytical subset), a sensitivity analysis using a binary outcome (mild vs moderate-or-severe) was performed. Residual diagnostic plots for all linear models are provided in the Supplementary Materials (Supplementary Figures 1 and 2). Maps in the manuscript were created using QGIS 3.14.16-Pi (QGIS Development Team). Other illustrations were created with packages in R.

## Results

### Schematic of workflow

**Figure 2:**
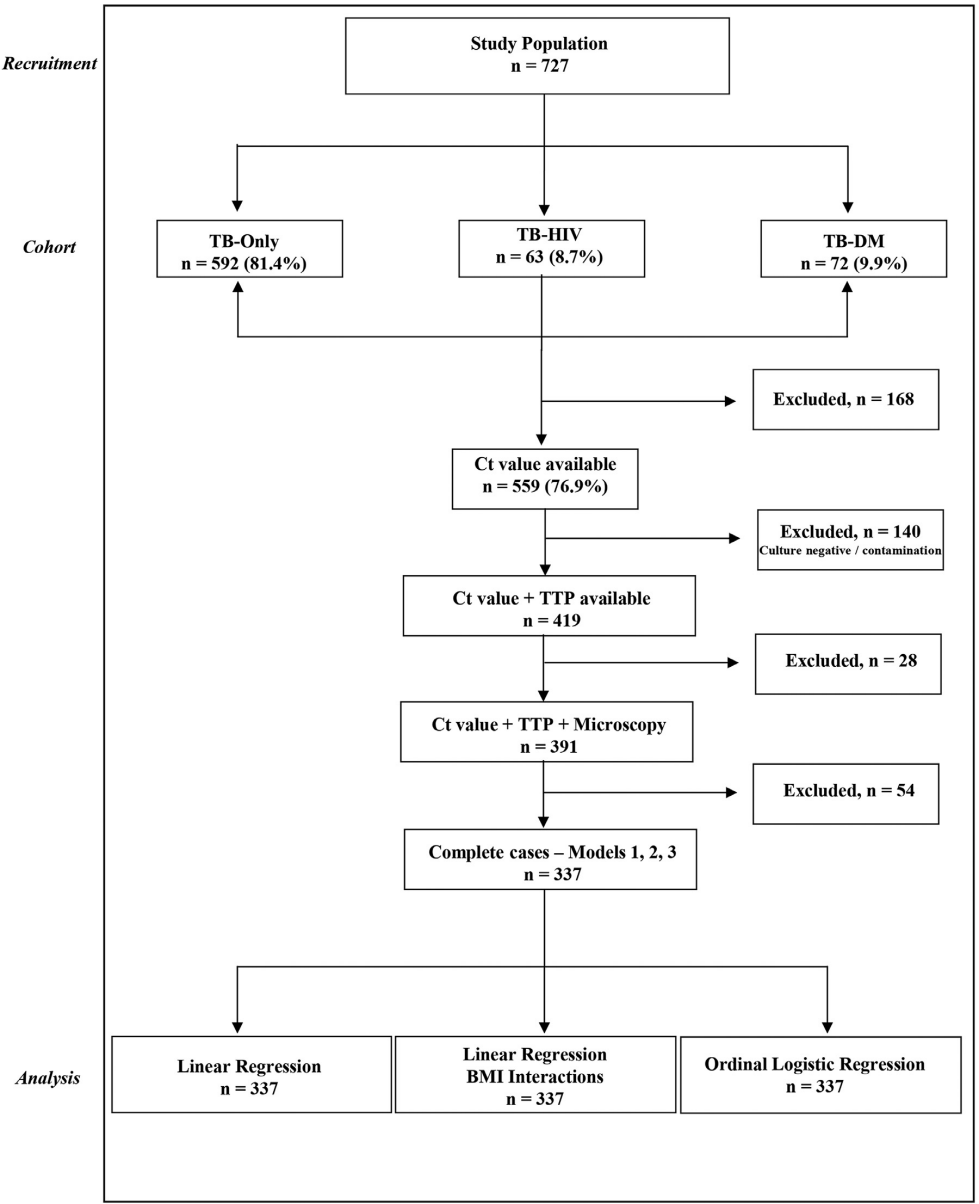
General schematic workflow for analysis.

A summary of the workflow from patient recruitment to laboratory analysis (Figure 2). A missing data table is also presented in Supplementary Table 1.

### Demographic and clinical characteristics

We first compared the demographic and clinical variables across cohorts. Seven hundred and twenty-seven (727) GeneXpert-confirmed TB cases were recruited as part of this study between October 2022 and December 2024. As expected per TB epidemiology in Ghana, the majority of the cases were men, 537 (73.9%), as compared with women (190, 26.1%), although the difference was not significant among the three cohorts (*P* = 0.0557): TB-only (592, 81.4%), TB-HIV (63, 8.7%), and TB-DM (72, 9.9%). However, women had higher proportions of TB-HIV (n = 23, 12.1%) and TB-DM (n = 23, 12.1%) than men. The median age of the participants was 42 years (interquartile range [IQR] 31-53). TB-HIV was more common in younger age groups (26-40 years), whereas TB-DM increased with age (41-70 years). Self-reported ethnicity differed significantly among participant groups (*P* < 0.0001), with the highest being Ewe, Ga, and Akan, respectively. The mean BMI for the participants was 19.37 kg/m^2^ (range: 8.8-40.0 kg/m^2^; median:18.68 kg/m^2^). The characteristics of the participants recruited are summarized in Table 1.

**Table 1:** Demographic and clinical characteristics of study participants.

Variable (Number analyzed)	Number, N (%)			Significance *P*-value
	TB-only	TB-HIV	TB-DM	
Demographic/Epidemiological Data				

**Gender (727)**				
Male (537)	448 (83.4)	40 (7.4)	49 (9.2)	0.0557
Female (190)	144 (75.8)	23 (12.1)	23 (12.1)	
**Age (726)**				
15-25 (119)	109 (91.6)	8 (6.7)	2 (1.7)	<0.0001
26-40 (214)	174 (81.3)	29 (13.6)	11 (5.1)	
41-70 (366)	284 (77.6)	26 (7.1)	56 (15.3)	
>70 (27)	24 (88.9)	0 (0.0)	3 (11.1)	
**Ethnicity (732)**				
Akan (152)	122 (80.3)	16 (10.5)	14 (9.2)	<0.0001
Ewe (277)	234 (84.5)	10 (3.6)	33 (11.9)	
Ga (187)	145 (77.5)	33 (17.6)	9 (4.8)	
Hausa (33)	23 (69.7)	5 (15.2)	5 (15.2)	
Guan (10)	6 (60.0)	0 (0.0)	4 (40.0)	
Others (73)	60 (82.2)	5 (6.8)	8 (11.0)	
**Marital status (695)**				
Married (250)	191 (76.4)	20 (8.0)	39 (15.6)	0.0002
Single (308)	268 (87.0)	28 (9.1)	12 (3.9)	
Divorced (94)	71 (75.5)	11 (11.7)	12 (12.8)	
Widowed (43)	31 (72.1)	4 (9.3)	8 (18.6)	
**Education (697)**				
Primary (107)	86 (80.4)	9 (8.4)	12 (11.2)	<0.0001
Secondary (172)	142 (82.6)	18 (10.5)	12 (7.0)	
Tertiary (59)	53 (89.8)	2 (3.4)	44 (6.8)	
None (93)	67 (72.0)	12 (12.9)	14 (15.1)	
**Occupation (686)**				
Skilled (211)	177 (83.9)	18 (8.5)	16 (7.6)	0.0112
Unskilled (329)	248 (75.4)	39 (11.9)	42 (12.8)	
Unemployed (149)	130 (87.2)	6 (4.0)	13 (8.7)	
**Nationality (700)**				
Ghana (677)	545 (80.5)	62 (9.2)	70 (10.3)	
Nigeria (6)	5 (83.3)	1 (16.7)	0 (0.0)	
Niger (9)	9 (100.0)	0 (0.0)	0 (0.0)	
Mali (4)	3 (75.0)	0 (0.0)	1	
Other African countries (4)	4 (100.0)	0 (0.0)	0 (0.0)	
**Smoking (655)**				
Yes (229)	193 (84.3)	19 (8.3)	17 (7.4)	0.2073
No (426)	336 (78.9)	42 (9.9)	48 (11.3)	
**Drug usage (656)**				
Yes (91)	80 (87.9)	8 (8.8)	3 (3.3)	0.0889
No (565)	453 (80.2)	53 (9.4)	59 (10.4)	
**Alcohol use/abuse (633)**				
Yes (87)	76 (87.4)	8 (9.2)	3 (3.4)	0.0967
No (546)	436 (79.9)	51 (9.3)	59 (10.8)	
**Clinical History**				
**TB History (703)**				
New case (601)	485 (79.9)	51 (9.3)	65 (10.8)	0.4398
Relapse (70)	58 (82.9)	7 (10.0)	5 (7.1)	
Return after default (26)	22 (84.6)	4 (15.4)	0 (0.0)	
Failure of Previous Treatment (6)	4 (66.7)	1 (16.7)	1 (16.7)	
**Body mass index (593)**				
Normal (316)	232 (73.4)	38 (12.0)	46 (14.6)	0.0002
Abnormal (277)	241 (87.0)	19 (6.9)	17 (6.1)	
**Cough (727)**				
Extreme (489)	391 (80.0)	56 (11.4)	42 (8.6)	0.0005
Mild (127)	104 (81.9)	6 (4.7)	17 (13.4)	
None (111)	98 (88.3)	0 (0.0)	13 (11.7)	
**Cough duration (134)**				
<2 weeks (9)	6 (66.7)	1 (11.1)	2 (22.2)	0.0658
≥2 weeks (125)	113 (90.4)	6 (4.6)	6 (4.6)	
**Chest pain (727)**				
Yes (531)	430 (81.0)	54 (10.2)	47 (8.9)	0.0248
No (196)	162 (82.7)	9 (4.6)	25 (12.8)	
**Dyspnea (727)**				
Yes (336)	256 (76.2)	47 (14.0)	33 (9.8)	<0.0001
No (391)	336 (85.9)	16 (4.1)	39 (10.0)	
**Hemoptysis (727)**				
Yes (74)	58 (78.4)	6 (8.1)	10 (13.5)	0.5467
No (653)	534 (81.8)	57 (8.7)	62 (9.5)	
**Night Sweat (727)**				
Yes (416)	333 (81.8)	41 (8.7)	42 (9.5)	0.3958
No (311)	259 (80.0)	22 (9.9)	30 (10.1)	
**Weight loss (727)**				
Yes (585)	469 (80.2)	62 (10.6)	54 (9.2)	0.0006
No (142)	123 (86.6)	1 (0.7)	18 (12.7)	
**Myalgia (727)**				
Yes (170)	130 (76.5)	22 (12.9)	18 (10.6)	0.0654
No (557)	462 (82.9)	41 (7.4)	54 (9.7)	
**Fever (727)**				
Yes (183)	141 (77.0)	23 (12.6)	19 (10.4)	0.0850
No (544)	451 (82.9)	40 (7.4)	53 (9.7)	

Significance calculated with Pearson’s χ².

DM, diabetes mellitus; HIV, human immunodeficiency virus; TB, tuberculosis.

### Distribution of bacterial load and disease severity

#### Bandim TB scoring and disease severity

**Figure 3:**
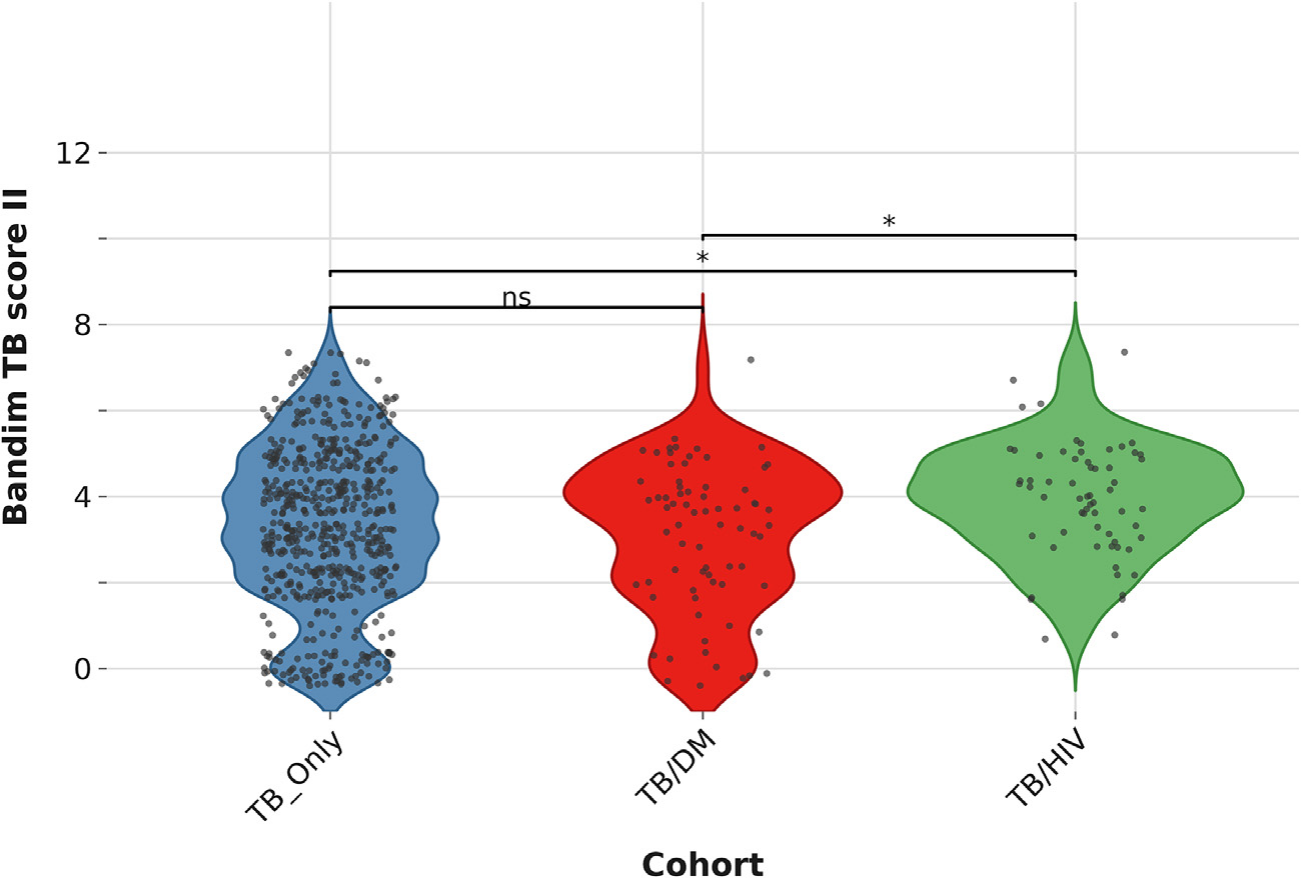
Distribution of Bandim TB score points across the different cohorts. Violins show the full score distribution; overlaid points represent individual participants. Significance brackets reflect pairwise Wilcoxon tests with Bonferroni correction (*P < 0.05; ns = not significant). Overall group difference: Kruskal-Wallis H = 8.64, *P* = 0.013.

The distribution of disease severity ranged from 0-7 (median = 3), indicating predominantly mild disease at the time of diagnosis. Severity distribution was mild (SCI = 523, 71.9%), moderate (SCII = 185, 25.4%), and severe (SCIII = 19, 2.6%). Bandim TB score II differed significantly across the three cohorts (Kruskal-Wallis H = 8.64, df = 2, *P* = 0.013). Post-hoc pairwise comparisons with Bonferroni correction revealed that TB-HIV participants had significantly higher severity scores than both TB-only (*P*.adj = 0.024) and TB-DM participants (*P*.adj = 0.012). No significant difference was observed between TB-only and TB-DM (*P*.adj = 1.000) (Figure 3).

#### Correlation between cycle threshold and Bandim score II

GeneXpert Ct values were available for 559 of 727 participants (76.9%); the remaining 168 were excluded due to the unavailability of Ct values at the time of analysis. The overall median Ct value was 18.94 (IQR: 18.16-21.04), reflecting moderate-to-high bacterial burden across the cohort. Ct values differed significantly across the three cohorts (Kruskal-Wallis H = 10.41, df = 2, *P* = 0.006). TB-HIV participants had the highest Ct values, indicating the lowest detectable bacterial burden: median 20.04 (IQR: 18.80-24.46), compared with TB-only (median 18.94, IQR: 18.16-21.04) and TB-DM (median 18.72, IQR: 18.03-20.59). Pairwise comparisons with Bonferroni correction confirmed that TB-HIV differed significantly from both TB-only (W = 8,735, *P* = 0.010) and TB–DM (W = 1,052, *P* = 0.009), while TB-only and TB-DM did not differ (W = 14,502, *P* = 0.917). This pattern is consistent with paucibacillary disease in HIV co-infection, in which immunosuppression alters sputum bacterial concentration and reduces GeneXpert yield independent of clinical severity.

Despite this group-level difference in bacterial burden, Ct value did not correlate with clinical severity as measured by Bandim TB score II across the full cohort (Spearman ρ = −0.006, *P* = 0.894, n = 559). This finding was consistent within each subgroup: TB-only (ρ = −0.047, *P* = 0.326), TB-DM (ρ = 0.144, *P* = 0.272), and TB-HIV (ρ = 0.074, *P* = 0.602). When participants were stratified by severity class (mild, moderate, severe), median Ct values were comparable across strata, confirming that bacterial burden as measured by GeneXpert does not reliably track clinical disease severity at presentation (Supplementary Figure 3, Supplementary Table 2).

#### Smear Microscopy and TTP across cohorts

Microscopy results across the three cohorts indicated a significant difference (χ² = 20.91, *P* = 0.007) in the distribution across the groups (Table 2). TB-DM group showed a higher proportion of 1+ smear positivity (41.8%) compared with TB-only (23.4%) and TB-HIV (22.6%), while high-grade positivity (3+) was most frequent in TB-only participants (12.2%) (Table 2). TB-HIV cases were more likely to be scanty or negative, consistent with paucibacillary disease, whereas the TB-DM group, although smaller in number, showed a high number of patients in the 1+ category.

**Table 2:** Microscopy results across the cohorts. Significance calculated with Pearson’s χ².

Microscopy Grade	Cohorts			*P*-value
	TB-only (%)	TB-HIV (%)	TB-DM (%)	
**3+**	67 (12.2)	3 (4.8)	5 (7.5)	**0.007**
**2+**	83 (15.1)	7 (11.3)	5 (7.5)	
**1+**	128 (23.4)	14 (22.6)	28 (41.8)	
**Scanty**	36 (6.6)	9 (14.5)	3 (4.5)	
**Negative**	234 (42.7)	29 (46.8)	26 (38.8)	
**Total**	**548**	**62**	**67**	

DM, diabetes mellitus; HIV, human immunodeficiency virus; TB, tuberculosis.

Time to positivity showed a small but significant negative correlation with Bandim TB score II across the full cohort (Spearman ρ = −0.125, *P* = 0.004, n = 531), indicating that shorter TTP, reflecting higher bacterial burden, was associated with more severe clinical disease. This association was statistically significant within the TB-only subgroup (ρ = −0.154, *P* = 0.001) but not among TB-DM (ρ = −0.055, *P* = 0.710) or TB-HIV participants (ρ = 0.105, *P* = 0.474), suggesting that the relationship between bacterial burden and clinical severity is modified by comorbidity status (Supplementary Figure 4).

### Mycobacterial infection across cohorts

**Figure 4:**
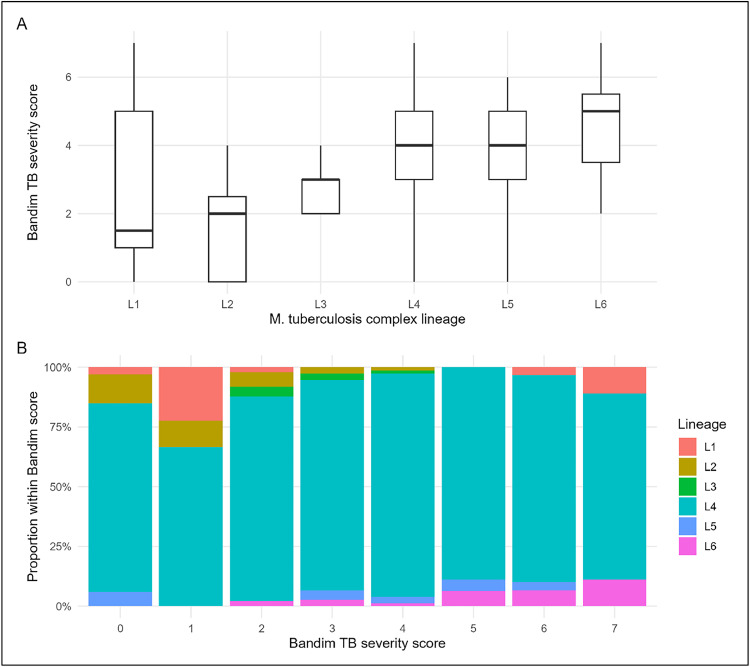
MTBC lineage distribution across Bandim TB score points. (a) Bandim TB severity scores plotted across MTBC lineages (L1-L6) using boxplots. L1 and L6 showed greater variability in severity, whereas L4 and L5 displayed a more concentrated distribution of scores. (b) Stacked proportional bar plot showing the relative contribution of each lineage within individual Bandim TB severity scores, highlighting lineage-specific patterns in clinical severity. MTBC lineage distribution across Bandim TB Score Points Kruskal-Wallis: H = 19.23, df = 5, *P* = 0.0017. MTBC, *Mycobacterium tuberculosis* complex; TB, tuberculosis.

Substantial diversity was observed among the circulating MTBC strains in this study. Most isolates were *Mycobacterium tuberculosis* sensu stricto (Mtbss) (n = 339, 93.9%) and *Mycobacterium africanum* (Maf) (n = 22, 6.1%). Species distribution did not differ across cohorts (p = 0.493); *M. tuberculosis sensu stricto* predominated in all three groups (TB-only 94.4%, TB-DM 89.7%, TB-HIV 93.3%). Overall lineage distribution did not differ significantly across cohorts (χ² = 17.44, df = 10, *P* = 0.065). Per-lineage analysis identified a borderline-significant difference for L5 (Maf), which was more prevalent in TB-DM (10.3%) than TB-only (2.6%) or TB-HIV ( p = 0.081) (Table 3). There was a significant difference (Kruskal-Wallis: H = 19.23, df = 5, *P* = 0.0017) when stratifying lineage distribution by Bandim TB scores, with most severe TB cases being infected with L4 (Figure 4).

**Table 3:** Lineages distribution across cohorts.

	TB-only n (%)	TB-DM n (%)	TB-HIV n (%)	Combined n	*P*-value
MTBC distribution	302	29	30	361	
**Species distribution**					
*M. tuberculosis*	285 (94.4)	26 (89.7)	28 (93.3)	339	0.493
*M. africanum*	17 (5.6)	3 (10.3)	2 (6.7)	22	
**Human-adapted MTBC lineages**					
Lineage_1	4 (1.3)	0 (0.0)	2 (6.7)	6	0.155
Lineage_2	9 (3.0)	2 (6.9)	0 (0.0)	11	0.332
Lineage_3	4 (1.3)	2 (6.9)	0 (0.0)	6	0.387
Lineage_4	268 (88.7)	23 (79.3)	26 (86.7)	317	0.267
Lineage_5	8 (2.6)	3 (10.3)	0 (0.0)	11	**0.081**
Lineage_6	9 (3.0)	0 (0.0)	2 (6.7)	11	0.409
**Lineage_4 sub-lineage distribution**					
Cameroon	169 (63.1)	13 (56.5)	16 (61.5)	198	0.603
Ghana	19 (7.1)	4 (17.4)	1 (3.8)	24	0.174
Haarlem	19 (7.1)	1 (4.3)	4 (15.4)	24	0.250
Uganda/Rwanda/SA	12 (4.5)	2 (8.7)	0 (0.0)	14	0.287
LAM	13 (4.9)	2 (8.7)	2 (7.7)	17	0.443
Others (New-1, X3)	19 (7.6)	1 (4.3)	2 (7.7)	22	1.000
Unclassified	15 (5.6)	0.(0.0)	1 (3.8)	16	

Significance calculated with Fisher’s exact test (Fisher-Freeman-Halton exact test). Percentages within MTBC distribution reflect proportions per cohort. L4 sub-lineage percentages calculated among d L4 isolates (n = 317). Individual Lineage 4 sub-lineage p-values were evaluated using a "sub-lineage X vs. other classified sub-lineages" matrix framework; unclassified Lineage 4 strains were excluded from the background comparison denominator to preserve statistical symmetry between the asymptotic and exact test models.

DM, diabetes mellitus; HIV, human immunodeficiency virus; LAM, Latin American-Mediterranean; MTBC, *Mycobacterium tuberculosis* complex; SA, South Africa; TB, tuberculosis.

These lineage differences warranted including the MTBC genotype as a covariate in subsequent multivariate models.

#### Determinants of TB clinical severity (Bandim score, main-effects model)

**Figure 5:**
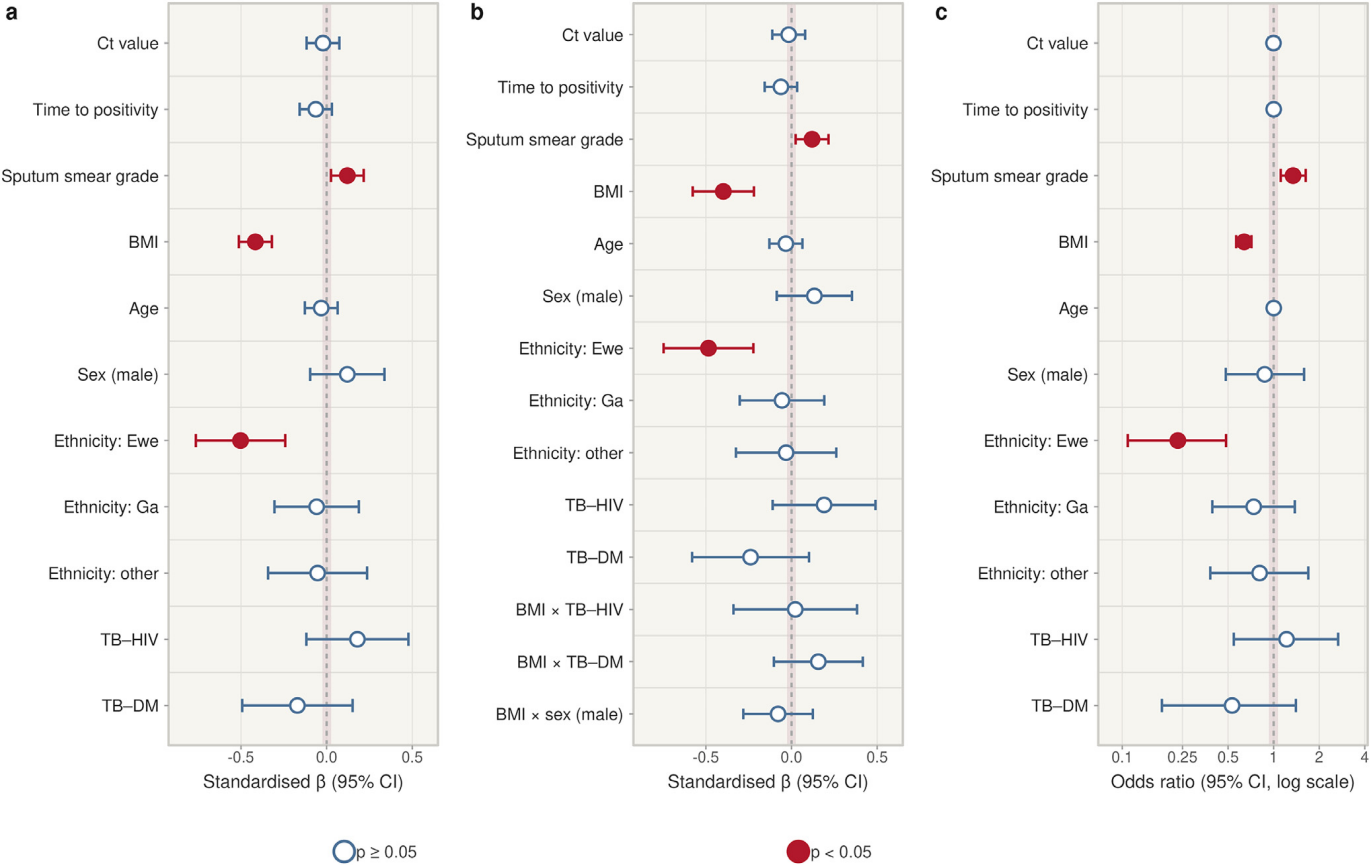
Multivariable regression analyses of predictors of TB severity and comorbidity. (a) Standardized linear regression showing independent predictors of Bandim TB score. Coefficients represent the SD change in severity per SD increase in each predictor. (b) Standardized linear regression including BMI interaction terms (BMI × TB-DM, BMI × TB-HIV, BMI × Sex). (c) Ordinal logistic regression showing odds ratios for classification into the high-severity group. ORs are displayed on a log scale. Across panels, points denote effect estimates and horizontal bars indicate 95% confidence intervals. red markers indicate statistically significant associations (*P* < 0.05). BMI, body mass index; DM, diabetes mellitus; HIV, human immunodeficiency virus; TB, tuberculosis.

We performed multivariable linear regression to identify independent predictors of TB severity using the Bandim TB score II. The model (n = 337) included bacterial (Ct, TTP, microscopy), and host factors (age, sex, BMI, comorbidity, ethnicity). We excluded lineage from the primary model, but a secondary model that incorporates it is presented in Section 3.4.4. Multicollinearity was assessed using the GVIF; all predictors had GVIF^1/(2·df)^ < 2.24, confirming no problematic multicollinearity (Supplementary Table 2). The overall model explained 17% of the variance in TB severity (adjusted R² = 0.170; F(11, 325) = 7.23, *P* < 0.001). BMI emerged as the strongest predictor of reduced disease severity (unstandardized β = −0.160 per 1 kg/m² increase; 95% CI −0.217 to −0.113; *P* = 7.29 × 10⁻¹⁶; standardized β = −0.416), indicating that undernutrition strongly contributes to clinical progression. Sputum smear microscopy grade was significantly associated with higher severity scores (unstandardized β = 0.139, 95% CI 0.029 to 0.249; *P* = 0.013; standardized β = 0.121), reflecting that higher bacillary burden in sputum corresponds to greater clinical severity. Ewe ethnicity was associated with lower Bandim scores compared with the Akan reference group (β = −0.800, 95% CI −1.216 to −0.385; *P* = 1.8 × 10^−4^; standardized β = −0.503). Ct value, TTP, age, sex, comorbidity, Ga ethnicity, and other ethnicity were not independently associated with severity after full adjustment (all *P* > 0.05; Figure 5a, Supplementary Table 2). To account for partial circularity, a new model was fitted, and BMI was removed from the TB score II outcome. BMI remained the key predictor for TB severity (standardized β = −0.240, 95% CI [−0.343 to −0.137], *P* < 0.001) (Supplementary Figure 5, Supplementary Table 3).

#### BMI-dependent effects on TB severity (interaction model, standardized coefficients)

To assess potential effect modification by BMI, we refitted the model including interactions between BMI and sex, TB-DM, and TB-HIV (n = 337). BMI remained independently protective (standardized β = −0.398, 95% CI −0.577 to −0.219; *P* = 1.6 × 10^−5^). None of the three interaction terms reached statistical significance: BMI×TB-DM (β = 0.157, 95% CI −0.103 to 0.416; *P* = 0.236), BMI × TB-HIV (β = 0.022, 95% CI −0.339 to 0.383; *P* = 0.905), and BMI × sex (β = −0.078, 95% CI −0.281 to 0.124; *P* = 0.448). Ewe ethnicity (β = −0.485 (95% CI −0.747 to −0.223, *P* = 0.00032) and smear microscopy (β = 0.120 *P* = 0.014) were also associated with lower Bandim TB scores (Figure 5b, Supplementary Table 4).

#### Predictors of mild, moderate and severe TB (ordinal logistic regression)

We used ordinal logistic regression to assess factors associated with increasing TB severity (Mild, Moderate, Severe) based on the Bandim TB score II categories (n = 337). Odds ratios (OR) represent the proportional odds of presenting with a more severe TB phenotype. The proportional-odds assumption was formally tested using the Brant test; the omnibus test showed no overall violation (χ² = 16.09, df = 11, *P* = 0.138). BMI showed a predictor-level violation (Brant *P* = 0.003), mechanistically explained by its structural contribution to the TB score II scoring system (BMI <18 kg/m² contributes one point, with an additional point for BMI <16 kg/m²); ordinal estimates for BMI are therefore interpreted alongside the binary sensitivity analysis below.

BMI remained the strongest predictor: each 1-unit increase in BMI was associated with a 36% reduction in the proportional odds of more severe TB (OR = 0.639, 95% CI 0.566-0.714; *P* = 3.7 × 10^−14^), consistent with undernutrition contributing to more severe disease. Microscopy positivity increased TB severity (OR = 1.343, 95% CI 1.113-1.627; *P* = 0.002). Ewe ethnicity was independently associated with lower odds of severe disease compared with the Akan reference group (OR = 0.233, 95% CI 0.109-0.485; *P* = 1.3 × 10^−4^). Ct value, TTP, age, sex, comorbidity, Ga ethnicity, and other ethnicity were not significant predictors (all *P* > 0.05; Figure 5c, Supplementary Table 5).

Given that the severe category comprised only 17 participants in the complete-case analytical subset (n = 19 in the full cohort), estimates at the upper severity threshold are interpreted descriptively. A sensitivity analysis using a binary outcome (mild vs moderate-or-severe, n = 337) yielded directionally consistent findings across all predictors (Supplementary Table 6), supporting the robustness of the primary ordinal model conclusions.

Excluding BMI from all the models did not materially alter the direction or significance of other predictors of disease severity.

#### Secondary ordinal model including lineages

After applying the same complete-case filter used in the primary models (225 participants TB-only: n = 178, TB-DM: n = 24, TB-HIV: n = 23) were available for a secondary ordinal logistic regression model that additionally included a binary lineage grouping (L4 vs Non-L4) as a covariate. In this subset, BMI remained the strongest predictor (OR = 0.622, 95% CI 0.527-0.723; *P* = 4.0 × 10^−9^), microscopy grade was significant (OR = 1.372, 95% CI 1.079-1.757; *P* = 0.011), and Ewe ethnicity was associated with substantially lower odds of more severe disease (OR = 0.188, 95% CI 0.068-0.497; *P* < 0.001). Non-L4 lineage was not significantly associated with severity in the ordinal model (OR = 1.106, 95% CI 0.427-2.773; *P* = 0.832). These findings support the primary model conclusions and confirm that the core associations are not an artifact of lineage confounding (Supplementary Table 7, Supplementary Figure 6, Supplementary Figure 7).

#### Comorbidity-specific associations

In multinomial logistic regression analysis comparing TB-DM and TB-HIV with TB-only cases, most demographic, clinical, and microbiological variables were not independently associated with comorbidity status. For TB-DM, increasing age was the only statistically significant independent predictor (aOR = 1.035, 95% CI 1.010-1.060; *P* = 0.005), with each additional year corresponding to a 3.5% increase in the odds of TB-DM comorbidity. No other predictor reached significance for this comparison, including BMI (aOR = 1.068, 95% CI 0.987-1.155; *P* = 0.101), Bandim TB score II (aOR = 0.824, 95% CI 0.630-1.077; *P* = 0.157), and bacterial load metrics (all *P* > 0.06). Higher Ct values were the only significant predictor for HIV comorbidity (aOR = 1.106, 95% CI 1.007-1.214; *P* = 0.036), with each unit increase in Ct value leading to 10.6% increased odds of TB-HIV comorbidity. All other factors, including Bandim TB score, bacterial burden, ethnicity, and lineage, were not significant predictors. (Supplementary Table 8, Supplementary Figure 8).

## Discussion

Bacterial load is widely considered a factor that can influence the severity and progression of the disease [14]. In TB, bacterial burden can be estimated using several approaches, including molecular methods such as GeneXpert, molecular bacterial load assay, which detects 16S ribosomal RNA copies, and sputum smear microscopy. This study sought to understand the drivers of heterogeneity in TB clinical presentation across different host contexts, including comorbid conditions such as HIV and diabetes. Using Bandim TB score II and bacterial load comparisons, alongside host and pathogen factors, we aimed to identify variables associated with disease severity at presentation. Our findings indicate that nutritional status, ethnicity and sputum smear grade were more consistently associated with disease severity than bacterial load, consistent with previous studies [[15], [16], [17]]. Although cycle threshold and other bacterial load metrics provide useful diagnostic information, they cannot be relied on as sole predictors of TB disease severity.

To further explore severity patterns, we compared Bandim TB scores across clinical cohorts, which showed a significant difference (*P* = 0.013), with TB-HIV individuals presenting with more severe symptoms, including dyspnea (14%), extreme cough (11.5%), and weight loss (98.4%). This is consistent with prior studies, where the low cluster of differentiation (CD)4 counts in patients with HIV exacerbate the symptoms observed through the disruption of granuloma integrity that leads to disseminated pathology [18]. This results in immune dysregulation, characterized by elevated inflammatory cytokines and impaired macrophage activation, which could drive symptom severity independent of bacterial load [19,20]. Importantly, TB-HIV participants were characterized by higher Ct values, reflective of lower detectable bacterial burden and higher Bandim TB scores compared with TB-only participants. This co-occurrence directly demonstrates the decoupling of molecular bacterial burden from clinical severity in immunocompromised individuals, representing the clearest evidence in this study that GeneXpert alone is insufficient for severity assessment in this population. Similarly, TB-DM morbidity was associated with altered clinical presentation, mostly in older patients. Hyperglycemia has been linked to reduced innate immune cell count, impaired neutrophil function, delayed antigen presentation, oxidative stress, and suboptimal Th1 immune responses, which may impair bacterial clearance and delay adaptive immune responses [21].

An important distinction, however, emerged, with sputum smear microscopy consistently associated with higher Bandim scores across the various models. These findings suggest that it is not bacterial burden per se, but high visible bacillary load specifically, that is associated with more severe clinical disease. This is consistent with studies showing that higher smear grade reflects greater bacillary burden and is associated with greater symptom severity and infectivity [22]. BMI emerged as the most significant associate of TB severity among all variables tested. However, the cross-sectional nature of this study precludes the determination of directionality, as reduced BMI may represent either a predisposing host vulnerability or a consequence of advanced disease at presentation. Lower BMI was consistently associated with increased severity, consistent with prior evidence linking malnutrition to poor TB outcomes and vice versa [23,24]. Cell-mediated immunity is severely weakened by undernutrition and impacts IFN-γ–mediated macrophage activation, impairing bacterial control [25]. In addition, malnutrition has been associated with increased expression of TB risk signatures and inflammation in contacts of TB patients, underscoring its role in disease progression [26]. Critically, BMI remained significantly associated with a modified Bandim score from which its scoring component had been removed (standardized β = −0.240, *P* < 0.001), confirming that this finding is not an artifact of partial score circularity but reflects genuine independent predictive value. Together, these findings support the importance of nutritional status in shaping TB severity and reinforce the rationale for integrating nutritional assessment and support in TB care, particularly in resource-limited settings.

The observed association between the Ewe ethnicity group and reduced TB severity, reflected by low Bandim scores, suggests that population-level factors may influence disease expression. This ethnic group has been previously associated with Maf infections, which are known to be associated with milder clinical presentations in West Africa [11,27]. Although host genetic variation has been shown to modulate TB susceptibility and clinical phenotype in other settings [28,29]; the present findings should be interpreted cautiously. In this context, ethnicity likely captures a composite of socio-cultural, environmental, and potentially biological factors rather than a single causal mechanism. Further genomic and immunological studies are needed to clarify the pathways underlying population-specific differences in TB severity.

MTBC lineages have been reported to differ in transmissibility and host immune interactions, potentially contributing to heterogeneity in clinical presentation across populations. In this cohort, lineage 4 predominated, consistent with West African epidemiology. Further exploratory analysis showed that lineage 2 infections were associated with milder disease at presentation compared to lineage 4 in this cohort. However, this finding is based on a few L2 isolates in the analytical subset and should be considered hypothesis-generating rather than conclusive. Although lineage 2 has been linked to severe disease in some Asian populations, the differing associations observed here may reflect population-specific host, environmental, or epidemiological contexts rather than intrinsic pathogenic differences [30]. Collectively, these findings support a model in which clinical severity is shaped by host–pathogen interactions, rather than pathogen lineage acting as an isolated determinant of disease severity.

The absence of a strong correlation between molecular bacterial burden and disease severity across cohorts contrasts with other studies, including that of Sarkar et al. [5], who observed a strong negative correlation between GeneXpert Ct values and the Bandim TB score. Sputum quality at the time of diagnosis can affect Ct values and cannot be used as a sole measure of bacterial load or TB severity. The discrepancy may additionally reflect differences in MTBC lineage distribution, host immunogenetic background, and healthcare-seeking patterns between Indian and Ghanaian populations, underscoring that the predictive value of Ct for severity may not be generalizable across settings. Instead, TB heterogeneity in clinical presentation appears to arise from an interaction of host immunity, metabolic health, and pathogen genotype. A major strength of this study is the integration of molecular bacterial burden metrics, standardized clinical severity scoring, comorbidity profiling, and MTBC lineage classification within a single multivariate analytical framework. Few studies in high-burden African settings have simultaneously evaluated host, pathogen, and clinical determinants of TB severity.

## Conclusion

TB severity in this Ghanaian cohort was more consistently associated with host factors such as nutritional status, ethnicity, and comorbidity, with GeneXpert bacterial load characterization playing a secondary role. These results further support the relevance of a host–pathogen interaction framework in understanding TB pathogenesis. Future studies should integrate host genomics, immunophenotyping and pathogen sequencing to understand the mechanistic pathway underlying these complex interactions. This can lead to improved identification of high-risk patients, earlier nutritional intervention, and more targeted clinical management.

## Limitations of the study

This study’s cross-sectional design can limit causal inferences. However, the large sample size, multicenter design and integration of molecular and clinical parameters strengthen the robustness of this study. Including patient treatment-outcome data could have strengthened the discussion. Additionally, incomplete anthropometric data for some participants may have influenced severity classification. Host factors such as BMI may reflect both pre-existing nutritional status and disease-related wasting. Longitudinal studies incorporating baseline pre-infection BMI or follow-up weight trajectories would be required to affirm causality. Self-reported ethnicity may act as a proxy for unmeasured confounders, including socioeconomic status, health-seeking behavior, nutritional patterns, or genetic background. Without direct measurement of these variables, residual confounding cannot be excluded.

## CRediT authorship contribution statement

**Theophilus Afum:** Conceptualization, Data curation, Formal analysis, Investigation, Methodology, Visualization, Writing – original draft, Writing – review & editing. **Prince Asare:** Methodology, Project administration, Supervision, Writing – review & editing. **Stephen Osei-Wusu:** Formal analysis, Project administration, Writing – review & editing. **Ivy Naa Koshie Lamptey:** Investigation, Writing – review & editing. **Susan Darkwahene-Boateng:** Investigation, Writing – review & editing. **Britta S. Meyer:** Data curation, Formal analysis, Writing – review & editing. **Tobias L. Lenz:** Funding acquisition, Supervision, Writing – review & editing. **Dorothy Yeboah-Manu:** Funding acquisition, Project administration, Resources, Writing – review & editing.

## Declaration of competing interest

The authors have no competing interest to declare.

## Appendix Supplementary materials

Image, image 1 dummy alt text

Image, image 2 dummy alt text

Image, image 3 dummy alt text

Image, image 4 dummy alt text

Image, image 5 dummy alt text

Image, image 6 dummy alt text

Image, image 7 dummy alt text

Image, image 8 dummy alt text

Image, application 9

Image, application 10

Image, application 11

Image, application 12

Image, application 13

Image, application 14

Image, application 15

Image, application 16

Image, application 17

Image, application 18

## Footnotes

Supplementary material associated with this article can be found, in the online version, at doi:10.1016/j.ijregi.2026.100937.
