# Supplementary material for: Host and pathogen factors modulate tuberculosis clinical severity independent of molecular bacterial burden: insights from a multicenter Ghanaian cohort
Source: IJID Reg. 2026 Jun 27;20:100937. doi: 10.1016/j.ijregi.2026.100937 (PMC13446365; doi:10.1016/j.ijregi.2026.100937)
Supplement: Supplementary file 1 [file mmc9.docx]

**Supplementary Figures**

**Figure S1.** Correlation between GeneXpert cycle threshold (Ct) value and Bandim TB score II across the three comorbidity cohorts. Upper panel: overall scatter plot with LOESS smoother (grey band, 95% CI) and linear fit (dashed red line). Lower panels: faceted by cohort (TB-Only, TB-DM, TB-HIV) with per-group Spearman correlation coefficient and p-value annotated. Spearman ρ = −0.006, p = 0.894, n = 559 complete pairs.

**Figure S2.** Correlation between time to positivity (TTP, days) and Bandim TB score II across the three comorbidity cohorts. Upper panel: overall scatter plot with LOESS smoother and linear fit. Lower panels: faceted by cohort with per-group Spearman ρ annotated. Overall Spearman ρ = −0.125, p = 0.004, n = 531 complete pairs.

**Figure S3.** Sensitivity analysis for BMI circularity: side-by-side forest plot comparing standardised regression coefficients (β, 95% CI) from the original TB score II model (left) and the modified TB score II model with the BMI scoring item removed (right). The BMI row is highlighted. BMI remained a significant independent predictor on the modified outcome (standardised β = −0.240, 95% CI −0.343 to −0.137, p < 0.001, n = 337).

**Figure S4.** Multinomial logistic regression: adjusted odds ratios (95% CI, log scale) for TB-DM vs TB-Only (left panel) and TB-HIV vs TB-Only (right panel). Filled circles indicate p < 0.05; hollow circles indicate p ≥ 0.05. Reference category: TB-Only. n = 337 complete cases.

**Figure S5.** Supplementary forest plot for the multivariable linear regression model incorporating individual MTBC lineages (n = 225). Panel A: unstandardised β coefficients (95% CI). Panel B: standardised β coefficients. Panel C: lineage-specific estimates with per-lineage sample sizes annotated. Non-L4 lineages are sparse (n = 4–9); estimates are presented descriptively.

**Figure S6.** Residual diagnostic plots for the main effects multivariable linear regression model (n = 337): Residuals vs Fitted values, Normal Q-Q plot, Scale-Location plot, and Residuals vs Leverage plot.

**Figure S7.** Residual diagnostic plots for the BMI interaction multivariable linear regression model (n = 337): Residuals vs Fitted values, Normal Q-Q plot, Scale-Location plot, and Residuals vs Leverage plot.

**Figure S8.** Residual diagnostic plots for the supplementary linear regression model incorporating individual MTBC lineages (n = 225): Residuals vs Fitted values, Normal Q-Q plot, Scale-Location plot, and Residuals vs Leverage plot.

**Supplementary Tables**

**Table S1.** Missing data summary for key analytical variables (n = 727). Columns show the number and percentage of participants with missing values for each variable and the reason for missingness. Variables with no missing data are included for completeness.

**Table S2.** Multivariable linear regression — main effects model (n = 337): unstandardised coefficients (β), standardised coefficients (β*), 95% confidence intervals, p-values, and generalised variance inflation factors (GVIF^(1/(2·df))). Outcome: Bandim TB score II (continuous). Reference levels: TB-Only (comorbidity), Female (sex), Akan (ethnicity). Adjusted R² = 0.170.

**Table S3.** Multivariable linear regression — BMI interaction model (n = 337): standardised coefficients (β*), 95% confidence intervals, and p-values for main effects and interaction terms (BMI × TB-DM, BMI × TB-HIV, BMI × sex). GVIF values reported; elevated values for interaction terms are expected by construction. Reference levels as in Table S2. Adjusted R² = 0.174.

**Table S4.** BMI circularity sensitivity analysis (n = 337): standardised regression coefficients (β*, 95% CI, p-value) from the original TB score II model and the modified TB score II model with the BMI scoring item removed, presented side by side for direct comparison. Modified score range: 0–6. Pearson r = 0.955 between original and modified scores.

**Table S5.** Ordinal logistic regression — primary model (n = 337): adjusted odds ratios (OR), 95% confidence intervals, and p-values for all predictors. Outcome: Bandim TB severity class (Mild ≤ 4 < Moderate ≤ 6 < Severe = 7). Brant test: omnibus χ² = 16.09, df = 11, p = 0.138 (no overall violation). BMI predictor-level violation: χ² = 8.80, p = 0.003 (mechanistically explained; see Section 2.7). Severe n = 17.

**Table S6.** Binary sensitivity analysis — Mild vs. Moderate-or-Severe (n = 337): adjusted odds ratios (OR), 95% confidence intervals, and p-values. Addresses the sparse Severe category (n = 17). Results are directionally consistent with the primary ordinal model across all predictors.

**Table S7.** Ordinal logistic regression — secondary model including MTBC lineage (n = 225): adjusted odds ratios (OR), 95% confidence intervals, and p-values. Includes binary lineage grouping (L4 vs. Non-L4) as an additional covariate. Whole-genome sequencing data available for 361 of 727 participants; complete-case n = 225 after applying primary model filters.

**Table S8.** Multinomial logistic regression — comorbidity determinants (n = 337): adjusted odds ratios (OR), 95% confidence intervals, and p-values for TB-DM vs. TB-Only and TB-HIV vs. TB-Only comparisons. Reference category: TB-Only. Pseudo R² = 0.112.

**Table S9.** Multivariable linear regression with individual MTBC lineages — supplementary model (n = 225): standardised coefficients (β*), 95% confidence intervals, and p-values. L4 is the reference lineage. Non-L4 lineage counts are sparse (L1: n = 4, L2: n = 4, L3: n = 5, L5: n = 9, L6: n = 7); estimates are presented for descriptive purposes and should not be over-interpreted. Adjusted R² = 0.282.
